# Myricitrin inhibited ferritinophagy-mediated ferroptosis in cisplatin-induced human renal tubular epithelial cell injury

**DOI:** 10.3389/fphar.2024.1372094

**Published:** 2024-06-07

**Authors:** Jiawen Lin, Yangyang Zhang, Hui Guan, Shuping Li, Yuan Sui, Ling Hong, Zhihua Zheng, Mingcheng Huang

**Affiliations:** ^1^ Department of Nephrology, Kidney and Urology Center, The Seventh Affiliated Hospital, Sun Yat-sen University, Shenzhen, China; ^2^ Department of Radiation Oncology, The First Affiliated Hospital of Zhengzhou University, Zhengzhou, China; ^3^ Department of Physiology, University of Oklahoma Health Sciences Center, Oklahoma City, OK, United States; ^4^ Molecular and Cellular Biology Laboratory, The Salk Institute for Biological Studies, La Jolla, CA, United States

**Keywords:** acute kidney injury, myricitrin, renal tubular epithelial cell, ferroptosis, ferritinophagy, oxidative stress

## Abstract

Cisplatin-induced acute kidney injury (AKI) increases the patient mortality dramatically and results in an unfavorable prognosis. A strong correlation between AKI and ferroptosis, which is a notable type of programmed cell death, was found in recent studies. Myricitrin is a natural flavonoid compound with diverse pharmacological properties. To investigate the protective effect of myricitrin against cisplatin induced human tubular epithelium (HK-2) cell injury and the underlying anti-ferroptic mechanism by this study. Firstly, a pharmacology network analysis was proposed to explore the myricitrin’s effect. HK-2 cells were employed for *in vitro* experiments. Ferroptosis was detected by cell viability, quantification of iron, malondialdehyde, glutathione, lipid peroxidation fluorescence, and glutathione peroxidase (GPX4) expression. Ferritinophagy was detected by related protein expression (NCOA4, FTH, LC3II/I, and SQSTM1). In our study, GO enrichment presented that myricitrin might be effective in eliminating ferroptosis. The phenomenon of ferroptosis regulated by ferritinophagy was observed in cisplatin-activated HK-2 cells. Meanwhile, pretreatment with myricitrin significantly rescued HK-2 cells from cell death, reduced iron overload and lipid peroxidation biomarkers, and improved GPX4 expression. In addition, myricitrin downregulated the expression of LC3II/LC3I and NCOA4 and elevated the expression of FTH and SQTM. Furthermore, myricitrin inhibited ROS production and preserved mitochondrial function with a lower percentage of green JC-1 monomers. However, the protection could be reserved by the inducer of ferritinophagy rapamycin. Mechanically, the Hub genes analysis reveals that AKT and NF-κB are indispensable mediators in the anti-ferroptic process. In conclusion, myricitrin ameliorates cisplatin induced HK-2 cells damage by attenuating ferritinophagy mediated ferroptosis.

## 1 Introduction

Acute kidney injury (AKI) is a critical medical illness with high mortality and morbidity, which is observed among approximately 5%–15% of hospitalized patients (Al-Jaghbeer et al., 2018; Martin-Cleary et al., 2021). AKI is clinically characterized by an abrupt decline in renal function. Known as a common cause of AKI, cisplatin is widely used as a chemotherapeutic drug in the management of solid malignant tumors (Tang C. et al., 2023). Nevertheless in prolonged, accumulating cisplatin could elicit renal dysfunction. The nephrotoxic responses to cisplatin not only limit its therapeutic potential but also affect the life quality of individuals with malignancies adversely.

Programmed cell death, including apoptosis and necrosis, has been identified as pivotal events in the pathological course of AKI, which manifests tremendous tubular cell damage in terms of histology (Sanz et al., 2023; Wu et al., 2022). As firstly proposed in 2012 by Dixon et al., ferroptosis is a form of novel programmed cell death placed on excessive iron production and overwhelming lipid peroxidation (Dixon et al., 2012). It has been showed in numerous studies that ferroptosis exists in both AKI patients and animal models established by different stimuli, such as LPS, cisplatin, ischemia-refusion, and folic acid (Bayır et al., 2023; Liang et al., 2022; Friedmann Angeli et al., 2014; Martin-Sanchez et al., 2017).

Iron metabolism is an essential biological activity responsible for ferroptosis. Generally, overloaded free iron could easily undergo the Fenton reaction, arousing massive oxidative damage to cell membrane lipid peroxidation (Fuhrmann and Brüne, 2022). Ferritinophagy is the process of autophagic degradation of ferritin and the release of extensive iron (Tang et al., 2018). While appropriate autophagy has emerged as a favorable cellular survival mechanism, excessive ferritin autophagy might be responsible for ferroptosis (Liu et al., 2020). It was found by Joseph D et al. that nuclear receptor coactivator 4 (NCOA4) was a specific receptor, which encouraged ferritin to be transported to the autophagosome and degraded eventually (Mancias et al., 2014). In brief, there is considerable intrigue in investigating the likely participation of NCOA4-mediated ferritinophagy in the progression of AKI.

Myricitrin, a bioactive component of bayberry, is a naturally occurring flavonoid with a diverse range of biological activities, which provides protections for multiple systems (Geng et al., 2023). Meanwhile, myricitrin exerts an immense impact on anti-oxidation and elimination of free radicals. Evidences provided by Sun et al. support the inhibitory effects of myricitrin on endothelial apoptosis induced by ox-LDL mediated ROS in the atherosclerosis mouse model (Qin et al., 2015). It has been demonstrated by animal studies that myricitrin can minimize liver I/R injury by preventing oxidative stress as well as inflammatory responses and boosting NO synthase activation (Shen et al., 2020). Currently, it was reported by Bin Zhao et al. that myricitrin attenuated cisplatin-induced kidney injury, mechanically eliminated reactive oxygen species, and inhibited apoptosis (Li et al., 2020). It is commonly recognized that reactive oxygen species triggers lipid peroxidation, which is the foundation of ferroptosis development (Pope and Dixon, 2023). However, there is a scarcity of published information concerning the anti-ferroptosis efficacy of myricitrin, whose mechanism of modulating AKI remains limitedly explored.

In the present investigation, we postulated that NOA4-mediated ferritinophagy contributed to ferroptosis in the context of cisplatin-induced renal tubular cells. Furthermore, we plan to investigate the potential role of myricitrin in the cell death of renal tubular cells and elucidate the underlying protective mechanism of myricitrin.

## 2 Methods

### 2.1 Reagent and antibody

The chemical reagents were purchased as below: Myricitrin (Myr): Selleck, S2327; Cisplatin (CDDP): MCE, HY-17394; 3-Methyladenine (3-MA): MCE, HY-19312; Chloroquine (CQ): MCE, HY-17589A; acetylcysteine (NAC): MCE, HY-B0215. All other chemicals were of high-grade purity available from commercial sources.

The antibodies were obtained as below: GPX4 (Abcam, ab125066), FTH (proteintech, 10727-1-AP), LC3A/B (CST, 4108), NCOA4 (abclonal, A5695), SQSTM1 (CST, 5,114), GAPDH (abclonal, AC001) and HRP-conjugated Goat Anti Rabbit IgG (proteintech, SA0001-2).

The relevant materials were provided as following: Cell Counting Kit-8 Kit (Beyotime, C0038), Annexin V/PI Apoptosis Analysis Kit (KeyGEN, KGA1013), Cell Ferrous Iron Colorimetric Assay Kit (Biosharp, BL1147A), Lipid Peroxidation MDA Assay Kit (Beyotime, S0131M), GSH Assay Kit (Jiancheng Bioengineering, A006-2-1), Bodipy 581/591 C11 (abclonal, RM02821), Reactive oxygen species Assay Kit (Beyotime, S0033S), MitoSox (ThermoFisher, M36008), and JC-1 applied to detect mitochondrial membrane potential (MCE, S0131M).

### 2.2 Construction of Myricitrin’s prediction targets network and GO function enrichment analysis

The SMILES format and structure of myricitrin were obtained from PubChem and we imported these information to SwissTargetPrediction database, comparative toxicogenomics database (CTD), and PharmMapper database (Daina et al., 2019; Davis et al., 2023; Wang et al., 2017). The gene interaction network was established by using STRING database (Szklarczyk et al., 2023). Furthermore, the identified genes were imported into Cytoscape to construct a protein-protein interaction (PPI) network for analysis by utilizing the CytoNCA method (Shannon et al., 2003; Tang et al., 2015). It is our aim to identify and evaluate the potential main targets of myricitrin in this study. In addition, the biological process of the key genes was enriched by using online Metascape database and visualized as a chordal graph by the application of bioinformatics (Zhou et al., 2019; Tang D. et al., 2023).

### 2.3 Pharmacodynamic mechanism analysis of myricitrin on cisplatin induced acute kidney injury

To explore the pharmacodynamic mechanism of myricitrin on acute kidney injury stimulated by cisplatin, the disease-related targets were collected at first from the following three databases: GenCards, Online Mendelian Inheritance in Man (OMIM) database, and DisGeNET (Stelzer et al., 2016; Amberger et al., 2015; Piñero et al., 2021). The two key phrases “cisplatin induced acute kidney injury” and “cisplatin nephrotoxicity” were used for searching, and only “*Homo sapiens*” proteins linked to the disease from results were selected. Finally, the targets were obtained from the overlaps between the myricitrin-related targets and the disease-related targets.

The “myricitrin on cisplatin acute kidney injury” perdition target network was constructed by using Cytoscape software. In the network, proteins were represented as nodes, and interactions between those molecular species were represented as edges. Functional annotation of target genes was analyzed by using the online Metascape database. The statistical significances were defined as *p* < 0.05 and the gene sets containing more than five genes were significant as well. Based on the online Metascape database, the GO and KEGG analysis projects were employed to explore the predicted action targets.

### 2.4 Cell culture

Human kidney epithelial tubular HK-2 cells were grown in F12 medium with 10% fetal bovine serum and 1% streptomycin/penicillin mixture. A humidified 5% CO2 atmosphere was applied to maintain all cell cultures at 37°C. After seeded in plates and cultured for 24 h, the HK-2 cells were incubated with fresh complete culture medium, and exposed to different treatments.

### 2.5 Cells model establishment and treatment

To establish cisplatin (CDDP) activated cells, HK-2 cells were exposed to different concentrations of cisplatin (2.5 or 5 ug/mL) for 24 h. To explore whether ferritinophagy occurs and is involved, we first employed the autophagy inhibitor, 3-MA (5, 10 uM) and CQ (10, 20 uM), to the HK-2 cells with cisplatin co-treatment for 24 h. To observe the effect of myricitrin on cisplatin activated cells, HK-2 cells were divided into four groups: the normal control, the CDDP group exposed to 5 ug/mL cisplatin, the Myr group cultured in medium containing 5 uM myricitrin for 24 h, and the CDDP + Myr group incubated with 5 uM myricitrin for 1 h prior to cisplatin stimulation for sequentially 24 h. Then, known as the autophagy inducer, 100 nM rapamycin (Rapa) was applied to the HK-2 cells 30 min before the administration of both myricitrin and cisplatin.

### 2.6 Cell viability assays

Cell viability was measured by using the Cell Counting Kit-8 (CCK8) assay. Cells were inoculated in a 96-well plate with a density of 5,000 cells per well. After overnight incubation the cells were treated by cisplatin and myricitrin for 24 h described above. The cells were incubated for 4 h after we applied 10 ul CCK8 solution in each well. Finally, the absorbance value was measured at 450 nm by a microreader.

### 2.7 Annexin V-PI assays

To explore the apoptosis level, the HK-2 cells from treatment groups and controls were harvested for different experimental needs, and then incubated by Annexin V-FITC and PI in the dark for at least 10 min according to the manufacturer’s protocols. Afterward, flow cytometry was utilized to examine the cells. Early apoptotic cells were determined by counting the percentage of Annexin V+/PI− cells; progressed apoptotic cells were obtained by counting the percentage of Annexin V+/PI + cells; necrotic cells were detected by counting the percentage of Annexin V-/PI + cells, and Annexin V−/PI− cells were considered as surviving cells.

### 2.8 Measurement of iron content

According to the manufacturer’s guidelines, an iron assay kit was employed to quantify the iron content in the HK-2 cells from treatment groups and controls. Briefly, cells were harvested and further lysed in iron lysis buffer by ultrasound. After centrifuged at 12,000 rpm for 10 min, the supernatant mixed with the working reagent was incubated for 15 min at room temperature. A microreader was employed to detect the absorbance at a wavelength of 562 nm.

### 2.9 Assessment of malondialdehyde (MDA)

To investigate the MDA concentration, the HK-2 cells from treatment groups and controls were subjected to lysis by using Western & IP lysis buffer. In accordance with manufacturer’s instructions, the resulting lysate was centrifuged at 12,000 rpm for 10 min and then measured by MDA Assay Kit which implemented TBA approach. Thereafter, 532 nm absorbance was recorded by using a microplate reader.

### 2.10 Assessment of glutathione (GSH)

The HK-2 cells from treatment groups and controls were homogenized by using an ultrasonic crusher and centrifuged at 12,000 rpm for 10 min after they were gathered. Then, the supernatant was collected for the following examination of GSH level. The experiment involved the process of the addition of samples mixed with GSH working mixture in the 96-well plate, which was incubated for 5 min at room temperature. The absorbance was determined at the wavelength of 405 nm.

### 2.11 Lipid peroxidation assay

The evaluation of lipid peroxidation was conducted by incubating different samples with 10uM Bodipy 581/591 for 1 h at 37 C in the dark. After the incubation was completed, the cells were washed twice with PBS to eliminate any surplus dye. The phenomenon of oxidized Bodipy was noticed by using a fluorescence microscope.

### 2.12 Intracellular ROS assay and mitochondrial ROS assay

The intracellular ROS production was observed by using reactive species oxygen Assay Kit. The HK-2 cells from treatment groups and controls were incubated with 1:2000 DCFH-DA at 37°C for 15 min in the dark. Then, after being washed twice by PBS, the HK-2 cells were resuspended in stain buffer and measured immediately by flow cytometry. Mitochondrial ROS level was detected by Mitosox stained. After incubated with 5 uM MitoSox for 60 min, the HK-2 cells were observated in fluorescence microscope.

### 2.13 Measurement of mitochondrial membrane potential

The Mito Probe JC‐1 Assay Kit was used to detect the changes in mitochondrial membrane potential (MMP). The HK-2 cells from treatment groups and controls were collected and incubated in 10 uM JC-1 working solution for 30 min in the dark. Then, the cells were washed twice by staining buffer and analyzed by flow cytometry within 1 h. The red aggregation showed normal MMP, while the green monomer indicated a reduction in MMP due to mitochondrial dysfunction. Results were presented as the percentage of green monomer stained by JC-1.

### 2.14 Western blot

Western blotting was employed to detect the protein expression of GPX4, FTH, NCOA4, LC3, and SQSTM1 in the cells from treatment groups and controls. The proteins obtained from the lysate of HK-2 cells were employed in RIPA buffer on ice and then the concentration was quantified by adopting the BCA protein assay kit. After the addition of 15 µg of distinct samples into each individual well, the proteins were separated by using 10%–12% SDS-PAGE gel and transferred onto a PVDF membrane. The membrane was blocked for 2 h at room temperature in the 5% non-fatty milk. The membranes were sectioned into different portions based on the molecular weight of the target protein. Subsequently, the pieces were subjected to overnight incubation with primary antibody under the temperature condition of 4°C. After using a suitable secondary antibody for incubation, the immunoblots were eventually detected by super ECL reagent. All protein bands’ intensity was measured by ImageJ.

### 2.15 Statistical analysis

All experiments were conducted three to five times, with each repetition carried out independently. Data was expressed as mean + standard deviation and analyzed by using GraphPad Prism 8. Data analysis and comparison between groups used one-way ANOVA and two-tailed Student’s t-test. Values of *p* < 0.05 were considered statistically significant.

## 3 Results

### 3.1 Screening and analysis of potential targets of myricitrin

**FIGURE 1:**
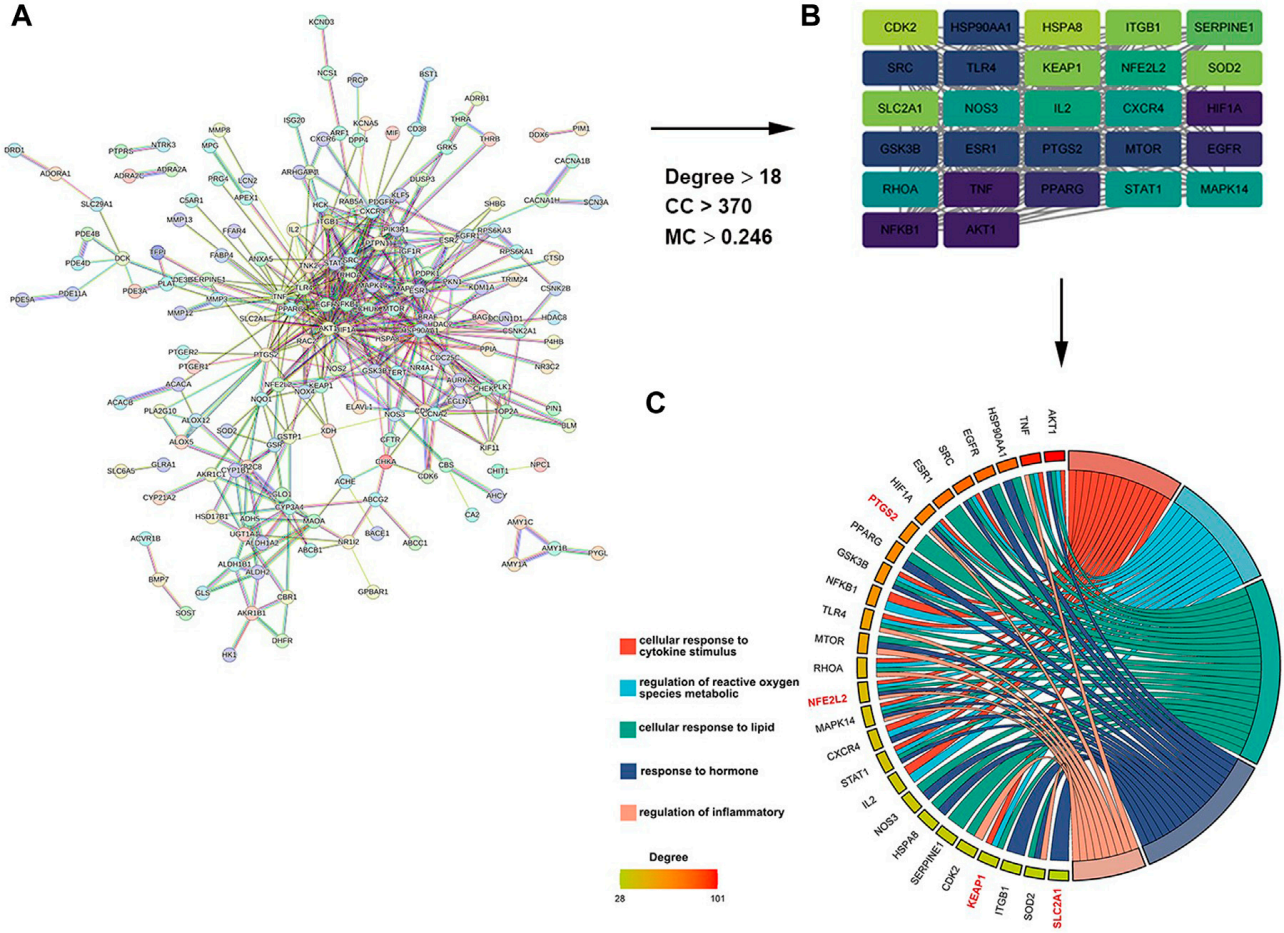
Screening and analysis of potential targets of myricitrin. **(A)** The Protein Interaction (PPI) network of myricitrin’s targets was gathered from the String database. **(B)** Network diagram of the most important targets of the myricitrin’s potential genes was made by using CytoNCA calculation. **(C)** Top enriched GO process of myricitrin’s targets was acquired from Metascape database, and the chordal diagram was constructed by representing enrichment items and their relevant genes.

As shown in Figure 1A, a total of 242 target genes of myricitrin were obtained by searching three online databases (PharmMapper database, SwissTargetPrediction database, and superpre database). With the help of the STRING database, a pharmacological target of the myricitrin network was constructed. According to Cytoscape by applying CytoNCA calculation, Figure 1B showed that a network of top 27 interactional genes is obtained by setting parameters (Degree ≥18, Betweenness Centrality ≥370, Closeness Centrality ≥0.248). Then, with analysis results of the 27 genes via Metascape database, it was surprisingly noted that the biological process of the top gene was mainly enriched in cellular response to cytokines stimulus, regulation of reactive oxygen species metabolic, cellular response to lipid, and so on. In addition, it was fascinating to mention that when conducting a search in the ferroptosis database and the related literature, a significant majority of the top 27 genes were identified as hallmark indicators of ferroptosis. These genes contain PTGS2, NFE2L2, KEAP1, SLC2A1, and so on (Figure 1C). To sum up, it can be speculated that administration of myricitrin was intricately countering the incidence and progression of ferroptosis.

### 3.2 Ferroptosis occurs on cisplatin induced renal tubular cells

**FIGURE 2:**
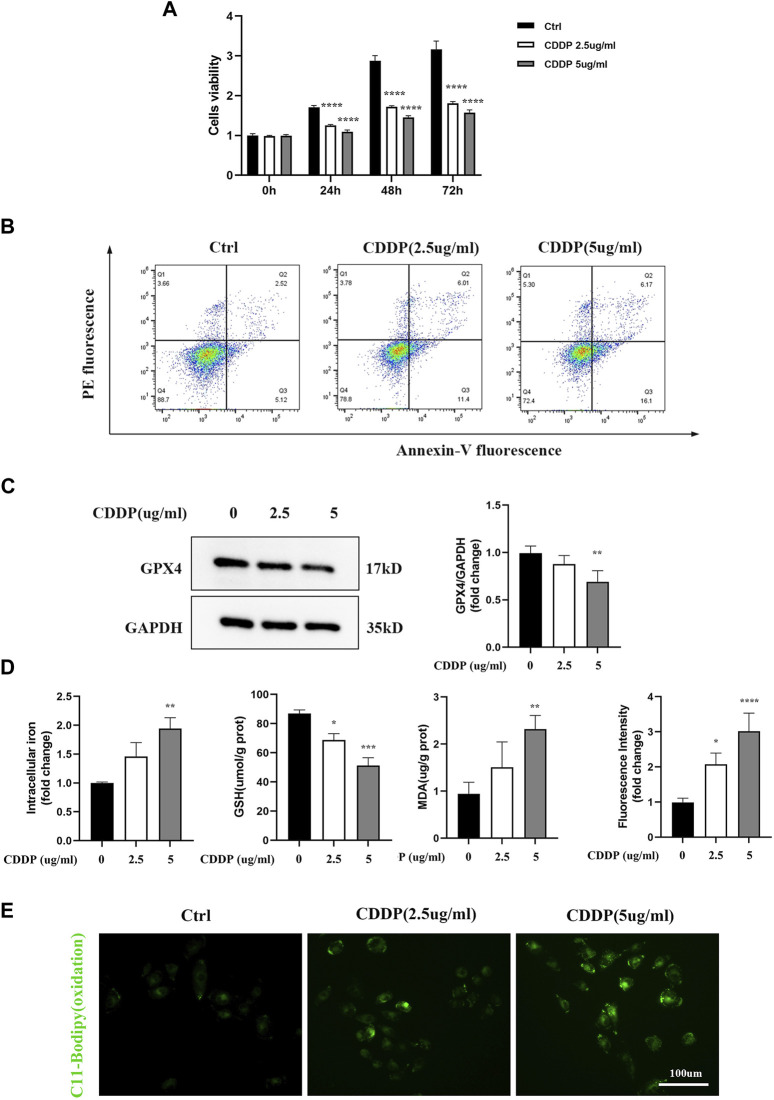
Renal tubular cells induced by Ferroptosis occurs on Cisplatin. *In vitro*, HK-2 cells were treated with different concentrations of cisplatin (0, 2.5, 5ug/mL). **(A)** Cell viability of HK-2 cells was determined by CCK-8 assays. **(B)** Annexin V-PI assay was used to determine the apoptosis cells. **(C)** Western blot was used to examine the expression of ferroptosis-related proteins GPX4. Protein levels were measured by using densitometry and normalized with GAPDH. **(D)** Intracellular iron was examined by Colorimetric Assay Kits. MDA and GSH were examined and normalized with the protein concentration. **(E)** Oxidation C11 BODIPY staining of the cisplatin activation HK-2 cells (scale bar = 100um). Compared with the control group, Data were presented as mean ± SD. (n = 3–4). *p < 0.05, **p < 0.01, ***p < 0.005, ****p < 0.001.

HK-2 cells were treated with different doses and by different times of cisplatin to determine the exact concentrations that inhibiting the cell growth. As shown in Figure 2A, cisplatin significantly inhibited the cell viability compared with the control group. Furthermore, confirmed with the above results, Figure 2B depicted that cisplatin increased the cell death-rate, which was detected by Annexin-V flow cytometry based on dose dependence. Known as specific markers for ferroptosis, Western blot was used to examine the GPX4 protein expression. The results showed that GPX4, a crucial enzyme against lipid peroxidation and ferroptosis, was also suppressed in the cisplatin group (Figure 2C). Furthermore, our results showed that cisplatin exposure led to enhanced accumulation of intracellular iron content. Lipid peroxidation was considered as a key component involved in the cell death cascade driven by ferroptosis. The amount of MDA could reflect the degree of intracellular lipid peroxidation. It was observed that cisplatin could significantly increase MDA levels in HK-2 cells (Figure 2D). C11-BOPIDY staining showed that the level of oxidation C11-BODIPY was significantly increased in the cisplatin group compared with the control group (Figure 2E).

### 3.3 Ferritinophagy Contributes to ferroptosis in cisplatin induced renal tubular cells

**FIGURE 3:**
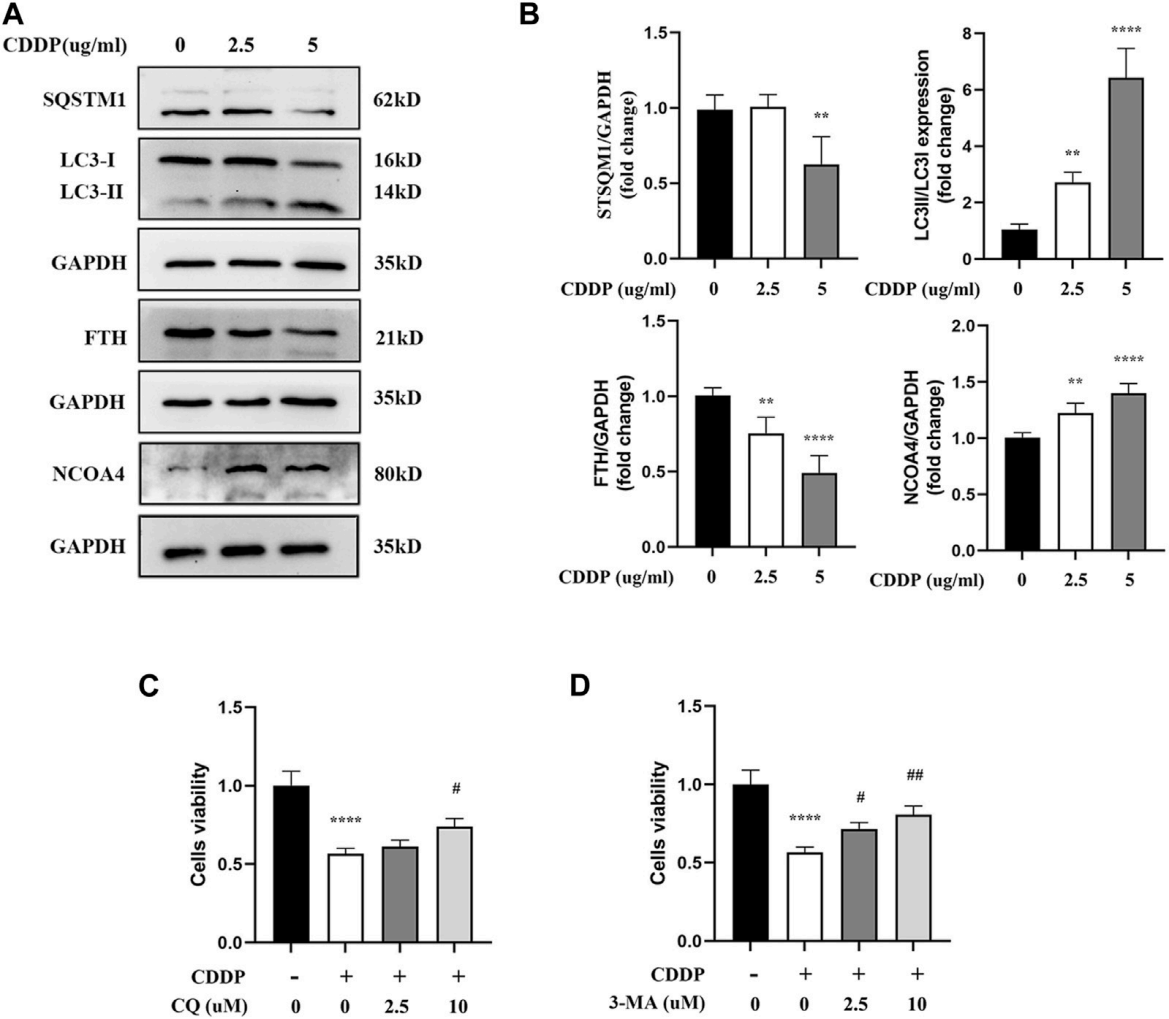
Ferritinophagy Contributes to Ferroptosis in Cisplatin induced Renal Tubular Cells. The HK-2 cell were stimulated with 2.5ug/mL or 5ug/mL cisplatin for 24 h. **(A)** The representative images of Western blot showed the expression of ferritinophagy-related proteins (LC3II/LC3I, FTH, NCOA4, SQSTM1) in cells (n = 4). **(B)** Protein levels were measured by using densitometry and normalized with GAPDH. **(C)**, **(D)** Cell viability of cisplatin activated HK-2 cells with co-incubation of different concentrations CQ (2.5, 10uM) and 3-MA (2.5, 10uM) was determined by CCK-8 assays (n = 3). Data were presented as mean ± SD. **p < 0.01, ****p < 0.001, compared with the control group and #*p* < 0.05, ##*p* < 0.01, compared with the CDDP group.

It has been confirmed in an increasing number of studies that autophagy participates in the occurrence and development of cisplatin-related renal tubular cell injury. Moreover, ferritinophagy is an autophagy mode of ferritin degradation mediated by NCOA4, which can regulate ferroptosis. Therefore, it was speculated that ferritinophagy might be involved in HK-2 cell injury caused by cisplatin. Meanwhile, it was speculated that NCOA4, FTH1, SQTM1, and LC3II/LC3I protein-related expression were obtained by applying Western blot. Compared with the CDDP group, the expressions of NCOA4 and LC3II/LC3I were downregulated in myricitrin pretreatment group, while the FTH1 and SQTMQ expression were increasing (Figures 3A,B). However, the classical autophagy inhibitors, specifically 3-methyladenine (3-MA) and chloroquine (CQ), were all employed to suppress the ferritinophagy activity. As illustrated in Figures 3C,D, high concentrations of 3-MA and CQ pretreatment could enhance the cell viability and diminish the cell damage. These results indicated that ferritinophagy might have participated in the progress of the injury of renal tubular cells with cisplatin administration.

### 3.4 Myricitrin significantly reduced ferroptosis upon cisplatin induction in renal tubular cells

**FIGURE 4:**
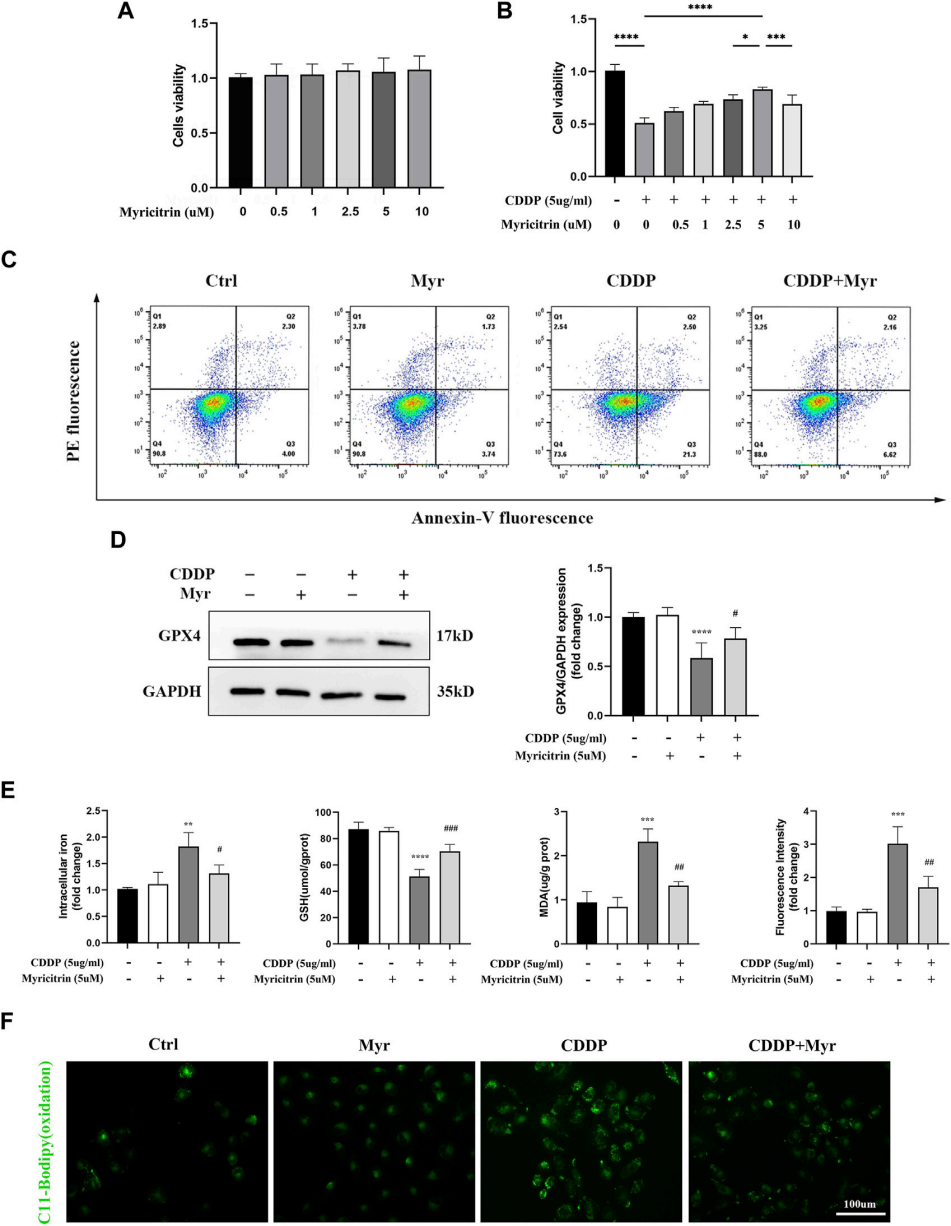
Myricitrin significantly reduced Ferroptosis upon cisplatin induction in renal tubular cells. **(A)** Cell viability of HK-2 cells treated with different concentrations of myricitrin (0, 0.5, 1, 2.5, 5, 10uM) for 24 h (n = 6). **(B)** Cell viability of cisplatin induced HK-2 cells treated with different concentrations of myricitrin (0, 0.5, 1, 2.5, 5, 10uM) for 24 h (n = 6). **(C)** The HK-2 cells were incubated with 5uM myritrin, 5ug/mL cisplatin or the combination for the following experiments in this section. Annexin V-PI assay determined the apoptosis cells. **(D)** Western blot was employed to examine the expression of ferroptosis-related proteins (GPX4). Protein levels were measured by using densitometry and normalized with GAPDH (n = 4). **(E)** Intracellular iron content was detected by Colorimetric Assay Kits. MDA and GSH, were examined and normalized with the protein concentration (n = 3–4). **(F)** Oxidation C11 BODIPY staining of the cisplatin activation HK-2 cells (n = 3), scale bar = 100um. Data were presented as mean ± SD. **p < 0.01, ***p < 0.005, ***p < 0.001, compared with the control group, and #*p* < 0.05, ##*p* < 0.01, ###*p* < 0.005, compared with the CDDP group.

First of all, according to the results of cell viability (Figure 4A), myricitrin at a concentration between 0.5 and 10 uM stimulation of 24 h showed no cytotoxicity effect on HK-2 cells. Therefore, 5 uM myricitrin incubation was selected for subsequent cell experiments. Secondly, we found that the decreased cell viability induced by cisplatin could be alleviated by myricitrin pretreatment (Figure 4B). In addition, it was observed that myricitrin could promote cell survival whereas cisplatin stimulated the apparent apoptosis of HK-2 cells (Figure 4C).

In Figure 4D, the HK-2 cells with myricitrin incubation showed a dramatic increase in GPX4 expression compared with the cisplatin induction group, which showed the depletion of GPX4. Meanwhile, the contents of intracellular iron in myricitrin treatment group were obviously lower than the cisplatin group (Figure 4E). As illustrated in Figure 4F, after myricitrin intervention, the increase of MDA content stimulated by cisplatin was partially reserved, whereas the levels of GSH inhibited by cisplatin were effectively restored. At the same time, the oxidation of C11-BODIPY fluorescent intensity was suppressed by myricitrin administration, indicating a decrease in lipid peroxidation and inhibition of ferroptosis, which was in alignment with the results mentioned before. In short, these results demonstrated that myricitrin ameliorated lipid peroxidation and ferroptosis against cisplatin-induced HK-2 cell injury.

### 3.5 Myricitrin attenuated ROS production and persevered mitochondrial function in cisplatin activated kidney tubular cells

**FIGURE 5:**
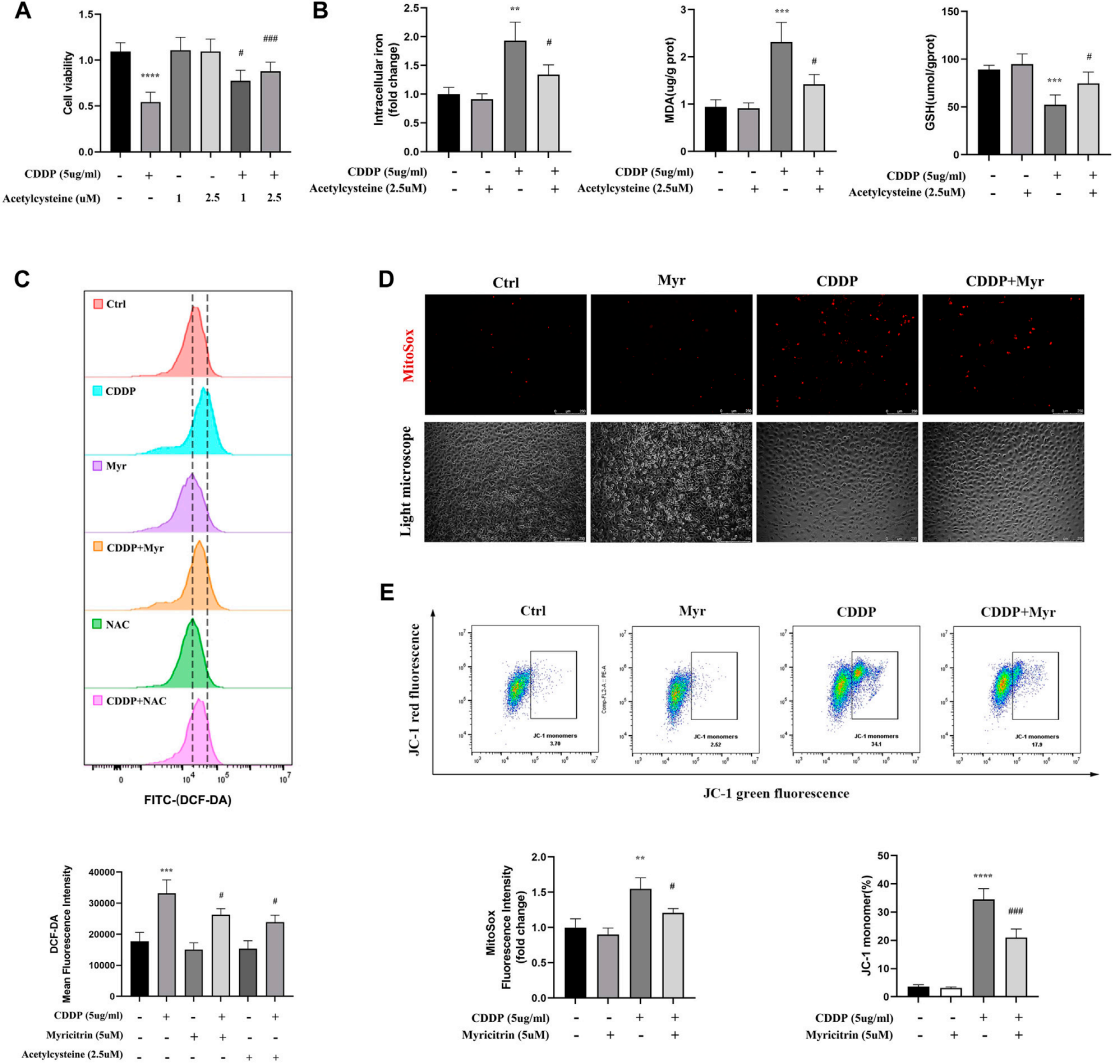
Myricitrin attenuated the ROS production and preserved the mitochondrial function. **(A)** CCK8 assays measured cell viability of HK-2 cells treated with indicated dose acetylcysteine or cisplatin or the combination for 24 h (n = 6). **(B)** Intracellular iron conten, MDA and GSH were detected in HK-2 cells incubated with 2.5uM acetylcysteine, or cisplatin, or the combination for 24 h (n = 4). **(C)** Cells were divided into six groups. HK-2 cells were stimulated with 2.5uM acetylcysteine or 5uM myritrin individually, and then treated with cisplatin for 24 h. Evaluation of ROS generation by using flow cytometry with DCFDA probe. The results were analyzed by quantification of mean fluorescence intensity (MFI) of DCFDA staining (n = 3). **(D)** Immunofluorescence analysis and quantification analysis of MitoSox in HK-2 cells (scale bar = 250um), the results were calculated by quantification of MFI (n = 3). **(E)** Flow cytometry was used to detect the mitochondrial membrane potential (Ψm) of HK-2 cells stained with JC-1. The dot plot illustrates the gate represented for JC-1 (green) monomer populations (n = 3). Data were presented as mean ± SD. ****p < 0.005, compared with the control group; ##*p* < 0.01, ###*p* < 0.005, compared with the CDDP group.

Previous research suggested the acetylcysteine, the oxidatve stress inhibitor, could attenuated the ferroptosis in various disease. The CCK-8 assays showed that does independed acetylcysteine could improved the cell viability of cisplatin induced cells (Figure 5A). Furthermore, intracellular iron content and MDA were lower in acetylcysteine group compare to the cisplatin group. Acetylcysteine could also increased the anti-oxidant GSH level (Figure 5B). In summary, we validated the positive role of ROS in the occurrence of ferrpotosis. To determine the effect of myricitrin on oxidative stress in renal tubular cells, the intracellular ROS levels were measured by using flow cytometry to detect the HK-2 cells stained with DCFDA. As shown in Figure 5C, ROS levels were significantly higher in cisplatin group, whereas both myricitrin and acetylcysteine could reverse oxidative stress via reducing intracellular ROS production. In addition, myricitrin could reduce the increased level of mitochondrial ROS, which induced by cisplatin (Figure 5D).

As the main organelle of energy metabolism and ROS production, mitochondria plays an important role in renal proximal tubular epithelial cells. In our study, it was found that cisplatin intervention in HK-2 cells resulted in a decrease in the percentage of JC-1 monomers, indicating MMP reduction and mitochondrial damage. However, lower percentage of green monomers was detected in the cisplatin-induced cells with myricitrin treatment (Figure 5E). These results manifested that myricitrin had an antioxidant effect as well as a protective function of mitochondria.

### 3.6 Myricitrin mitigated ferroptosis in cisplatin activated tubular epithelium cells via inhibiting NCOA4 mediated ferritinophagy

**FIGURE 6:**
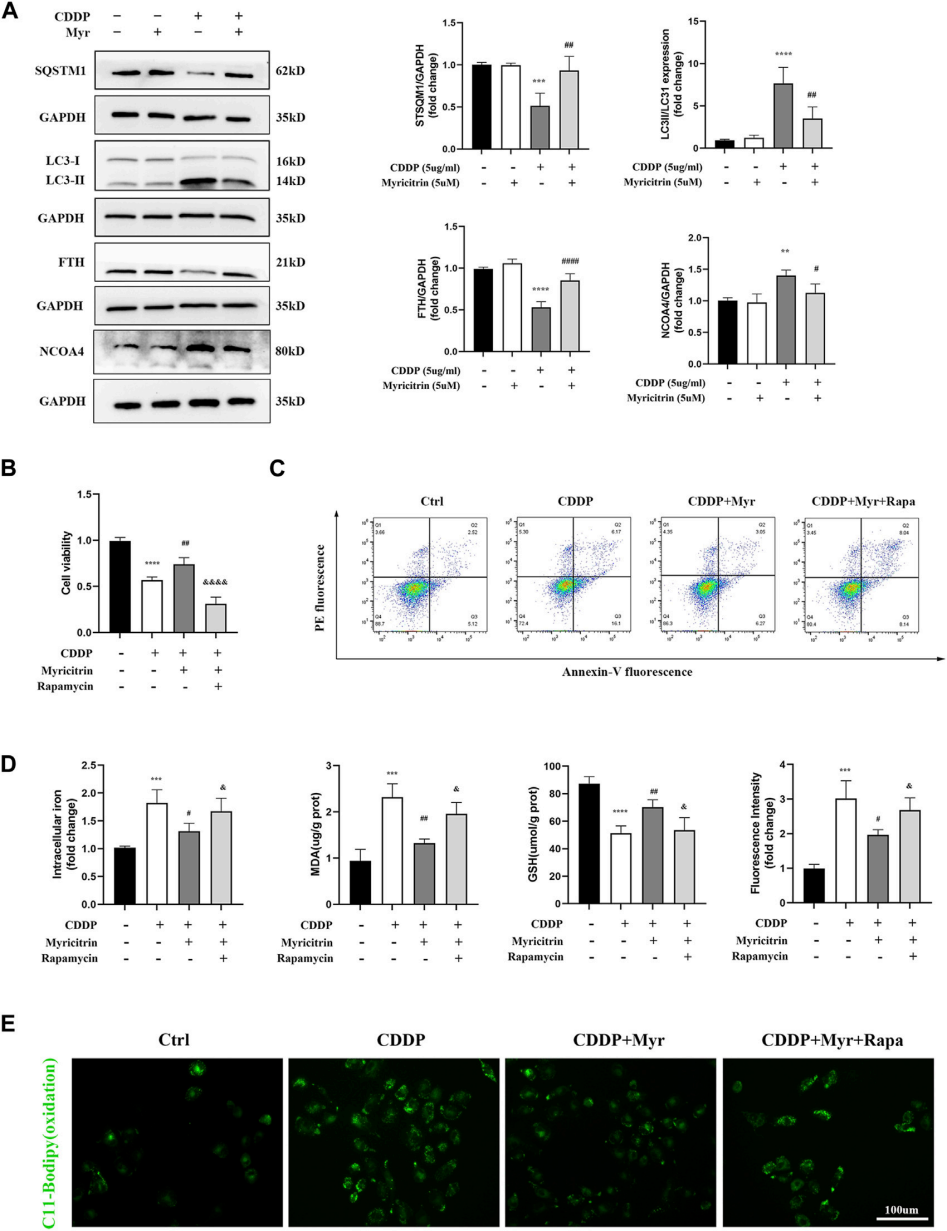
Myricitrin mitigated ferroptosis in cisplatin activated tubular epithelium cells via inhibiting NCOA4 mediated ferritinophagy. **(A)** Western blot examined the expression of ferritinophagy-related proteins (LC3II/LC3I, FTH, NCOA4, SQSTM1) from HK-2 cells treated with 5uM myricitrin (Myr), 5ug/mL cisplatin (CDDP), myricitrin + cisplatin (CDDP + Myr), or without both drugs (Ctrl) for 24 h. Protein levels were measured by using densitometry and normalized with GAPDH (n = 4). **(B)** After rapamycin appllied to HK-2 cells 30 min, both myricitrin and cisplatin were added to stimulated cells for 24 h. Cell viability of cisplatin activated HK-2 cells with co-treatment of rapamycin and myricitri (n = 5). **(C)** Annexin V-PI assay was used to determine apoptosis cells. **(D)** Intracellular iron content, MDA and GSH were examined and normalized with the protein concentration (n = 5). **(E)** Oxidation C11 BODIPY staining of the cisplatin activation HK-2 cells (scale bar = 100um). Data were presented as mean ± SD. *p < 0.05, **p < 0.01, ****p < 0.005, compard with the control group; #*p* < 0.05, ##*p* < 0.01, ###*p* < 0.005, compared with the CDDP group; &*p* < 0.05, &&&&*p* < 0.001, compared with the CDDP + Myr group.

It has been demonstrated in our earlier research that the autophagic degradation of ferritin, a process referred to as ferritinophagy, can promote ferroptosis in activated HK-2 cells with cisplatin stimulation. Additionally, NCOA4 is a specific cargo receptor that facilitates ferritin degradation in lysosomes. Herein, we investigated whether the NCOA4 mediated degradation of ferritin was associated with myricitrin’s protective mechanism against ferroptosis induced by cisplatin in renal tubular cells. Western blotting revealed that the expression of vital ferritinophagy makers LC3II/LC3I and NCOA4 were downregulated, while FTH as well as SQTM1 expression were upregulated by myricitrin treatment (Figure 6A).

To further investigate the role of ferritinophagy in cisplatin-induced renal tubular cell injury, activated HK-2 cells were pretreated with the ferritinophagy inducer and rapamycin (Rapa), and co-treated with myricitrin for 24 h. Our results demonstrated that myricitrin alleviated ferroptosis by promoting the cell viability and survival (Figures 6B,C), decreasing intracellular iron level and MDA content, and increasing GSH content, which was reversed by Rapa co-treatment (Figure 6D). In addition, results from C11-BODIPY immunofluorescence showed that myricitrin significantly decreased the level of lipid peroxidation, while co-incubation with Rapa deteriorated this influence (Figure 6E). Overall, these findings revealed that myricitrin suppressed the activated HK-2 cells ferroptosis by inhibiting NCOA4-mediated ferritinophagy.

### 3.7 Potential targets of the action targets of myricitrin on cisplatin induced acute kidney injury

**FIGURE 7:**
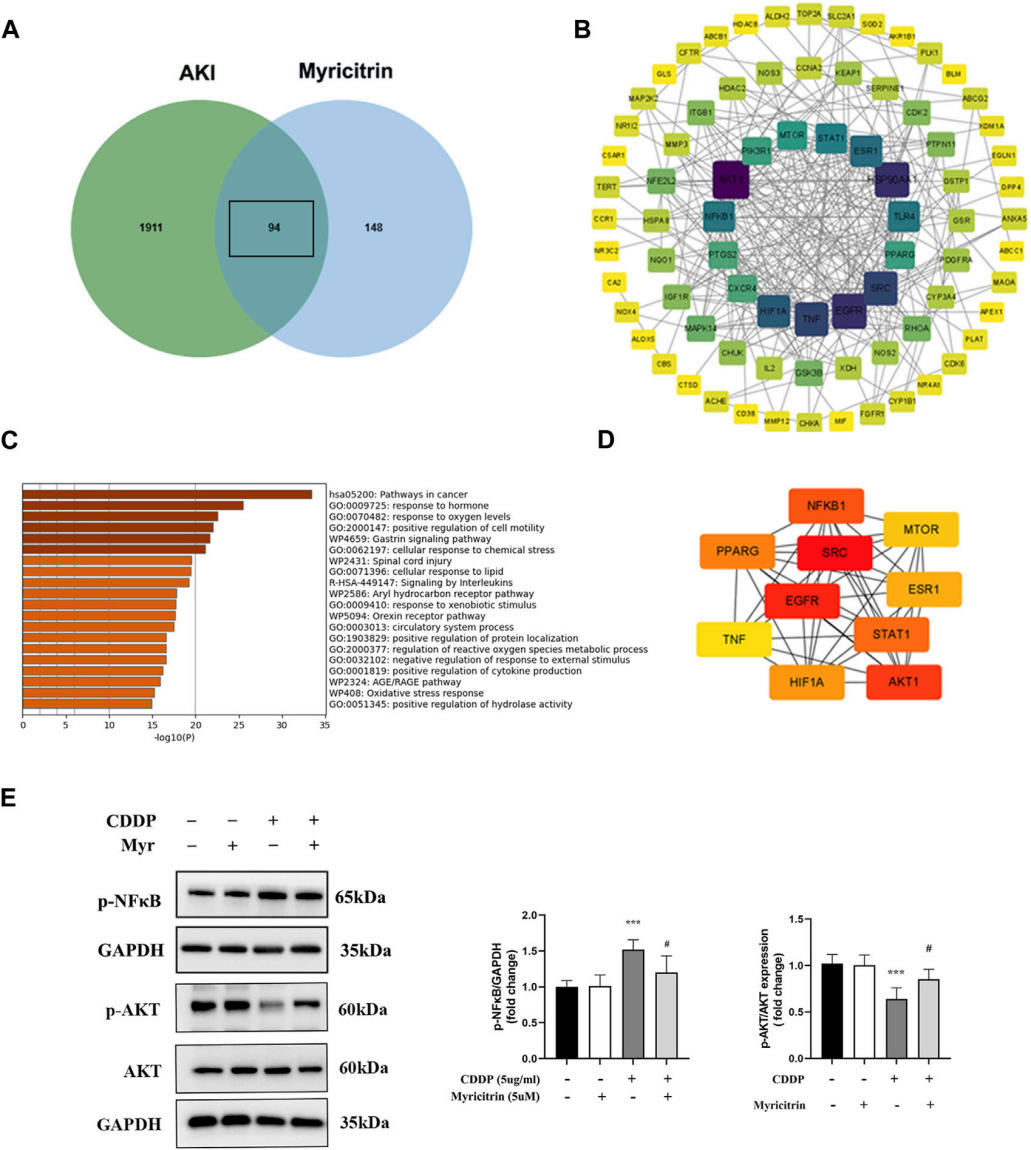
Potential targets of the action targets of Myricitrin on Cisplatin induced acute kidney injury. **(A)** Venn diagram illustrated the cross-section between cisplatin-induce acute kidney injury target and myricitrin target. **(B)** Network diagram of the predictive targets was constructed and visualized using Cytoscape software. **(C)** GO and KEGG pathway enrichment analysis were obtained from online Metascape database. **(D)** The 10 hubs genes were identified by utilizing CytoNCA algorithm. **(E)** The HK-2 cells were incubated with 5uM myritrin as monotherapy or in combination with 5ug/mL cisplatin for 24 h. Western blot showed the AKT and NF-κB protein phosphorylation expression of different group of HK-2 cells (n = 5). Data are presented as mean ± SD. ***p < 0.005, compare to the control group; #*p* < 0.05, compare to the CDDP group.

From GeneCards and OMIM, 2005 related targets for cisplatin-induced acute kidney injury were retrieved. Figure 7A displayed 94 putative targets for myricitrin acting on cisplatin induced acute kidney injury that derived from gene mapping. After removing the irrelevant genes from STRING database, the protein interaction network was created and enhanced by Cytoscape software. Figure 7B illustrated the construction of the targets of “myricitrin—cisplatin induced AKI” network. As shown in Figure 7C, the GO and KEGG pathway analysis results obtained from Metascape database, showed the enrichment in the process of response to lipid and reactive oxygen species metabolism, which was closely related to ferroptosis mechanism. Subsequently, the CytoNCA algorithm was utilized to identify the key proteins, including AKT1, SRC, EGFR, NFKB1, STAT1, PPARG, HIF1A, MTOR, ESR1, TNF, and so on (Figure 7D). Hence, it seemed sensible to assume that the AKT and NF-κB pathway might be implicated in myricitrin’s anti-ferroptosis effect on cisplatin-induced acute kidney injury.

## 4 Discussions

At present, our pharmacology network analysis demonstrated that myricitrin might display potential efficacy in preventing ferroptosis. In the meantime, it was observed that ferroptosis regulated by ferritinophagy participated in the damage of cisplatin activated HK-2 cells. Additionally, myricitrin significantly suppressed the cell damage by reducing ferroptosis and ferritinophagy, as well as preserving the mitochondrial function. In summary, these results revealed that inhibiting ferritinophagy-mediated ferroptosis might serve as the underlying mechanism of myricitrin against cisplatin-induced HK-2 cell injury.

Cisplatin is extensively employed as a chemotherapeutic drug for the treatment of solid tumors. With the rising use of this antineoplastic medication, acute kidney injury is known as one of the inevitable complications which deserves our attention. Recent studies have reported that inflammation, oxidative stress, vascular injury, endoplasmic reticulum stress, and tubular cell death are all involved in the pathogenesis of cisplatin-induced nephrotoxicity (McSweeney et al., 2021; Holditch et al., 2019).

Ferroptosis, an emerging type of regulatory cell death, is characterized by iron accumulation and excessive lipid peroxidation. There are mounting researches showing that ferroptosis plays a role in the field of renal disease, including diabetic nephropathy, renal fibrosis, and acute kidney injury caused by diverse factors (Wang Y. et al., 2022; Li et al., 2021; Tonnus et al., 2021; Wang J. et al., 2022). In our research, the renal proximal tubular epithelium cell was applied to establish the vitro models. Firstly, it was found that cisplatin could induce cell viability reduction and the process of cell death. Moreover, Fe2+ and lipid peroxidation accumulation were detected in HK-2 cells stimulated by cisplatin. GPX4 is recognized as a central repressor of ferroptosis in limiting lipid peroxidation (Seibt et al., 2019; Chen et al., 2023; Miao et al., 2023). The dysregulation of GPX4 is implicated in the ferroptosis execution. As western blotting illustrated, cisplatin reduced the expression of GPX4 expression of HK-2 cells, suggesting that ferroptosis contributes to cell injury related to cisplatin, aligning with earlier findings reported in the literature.

Intracellular iron homeostasis is critical for governing ferroptosis. Mechanistically, ferritinophagy facilitates the autophagic degradation of ferritin, resulting in iron release and Fe2+ dependent lipid peroxidation. NCOA4 is an essential factor regulating ferritinophagy by governing the autolysosome formation and ferritin degradation. It was observed that the protein level of FTH and SQTMQ1 dramatically decreased, while the NCOA4 protein level and the ratio of LC3II/LC3I increased in the presence of cisplatin. However, ferritinophagy inhibitor 3-MA or CQ significantly promoted cisplatin activated the cell viability. In these experiments, it had been discovered that ferritinophagy was reinforced in cisplatin activated HK-2 cells and this process ultimately led to ferroptosis condition.

Myricitrin is a flavonoid compound with several feasible medicinal activities, including regulating oxidative stress, inflammatory response, and apoptosis. More significant researches are required to explore the myricitrin’s molecular mechanisms. The pharmacology network method offers a systematic observation of the process of medications, focusing on the interaction network among disease, gene, and drug (Nogales et al., 2022). Therefore, we leveraged this approach and performed a GO enrichment study to evaluate the identified 27 hub genes obtained from network analysis. Interestingly, these genes exhibited enrichment in the biological process of regulating reactive oxygen species metabolic and cellular response to lipids, which was associated with ferroptosis. It is noteworthy that PTGS2, NFE2L2, KEAP1, and SLC2A1 are recognized as crucial biomarkers for ferroptosis, which suggest a promising potential for therapeutic intervention in mitigating this process. There have been reports about the myricitrin’s preventive effect on various kidney diseases (Dua et al., 2021; Weng et al., 2019). It was found by Zhao et al. that myricitrin played a protective role against cisplatin-induced acute kidney injury through the inhibition of oxidative stress and inflammation (Li et al., 2020). However, whether myricitrin could mitigate cisplatin-induced renal tubular epithelial cell impairment via inhibiting ferroptosis remains uncertain. In the following study, we explored the function of myricitrin in cisplatin-induced renal tubular epithelial cell damage. While 5uM myricitrin was administered as a pretreatment to cisplatin activated HK-2 cells, it was found that myricitrin greatly prevented the cell damage by reducing ferroptosis, with regards to the phenomenon of Fe2+, lipid peroxidation reduction, and GPX4 expression escalation compared with the model group. Along with the detection of ferritinophagy protein, it was found that myricitrin not only mitigated the expression of NCOA4 and the ratio of LC3II/LC3I, but it also improved FTH and SQTMQ1 levels. However, ferritinophagy inducer rapamycin co-incubation reversed the protection against ferroptosis mentioned above partially. In short, it was revealed in these results that the inhibition of ferritinophagy mediated ferroptosis might serve as the underlying mechanism of myricitrin against cell injury.

Oxidative stress is characterized by the overproduction of ROS and depletion of endogenous antioxidant defense function. Oxidative stress could exacerbate cellular metabolic homeostasis, and ultimately cause malfunctioning mitochondria to generate lower ATP (Forman and Zhang, 2021). Our study herein demonstrated that myricitrin attenuated the abundant ROS generation induced by cisplatin and maintained the mitochondrial function by preserving a higher level of MMP.

Finally, the pharmacological network analysis was applied to investigate the possible mechanism of how myricitrin influences renal tubular cells induced by cisplatin. The activation of AKT and NF-κB was determined to be an important target of myricitrin for cisplatin-related nephrotoxicity. AKT is widely recognized as a major autophagic blocker by exerting its influence on inactivating ULK1 and VPS34 complex, which are responsible for cellular digestion initiation (Yu et al., 2015; Zhang et al., 2020). Activation of the mTORC1 complex, epigenetic modification of FOXO, and direct regulation of autophagic protein are the main mechanisms of AKT silencing autophagy (Cheng, 2019; Bach et al., 2011). Interestingly, it has been shown in emerging evidences that activation of AKT could attenuate the effect of ferritinophagy modulation significantly. It was reported by Scott et al. that apolipoprotein E potently blocked the degradation of ferritin in murine mesencephalic neurons by stimulating the PI3K/AKT pathway and inducing AKT phosphorylation (Belaidi et al., 2022). Yi Cai et al. demonstrated that the activation of p38/AKT signaling in the host’s macrophages was the initiation of the ferritin degradation cascade provoked by *mycobacterium tuberculosis* (Dai et al., 2023). Besides, it was reported that autophagy was associated with NF-κB activation in acute kidney injury both *in vivo* and *in vitro* (Pan et al., 2021; Wu et al., 2015). In combination with the previous researches, we recognized myricitrin as an inhibitor against ferroptosis by conducting the phosphorylation modification of AKT and NF-κB.

## 5 Conclusion

This study presents novel findings which indicate the involvement of ferritinophagy in the process of ferroptosis in cisplatin-induced renal tubular cell damage. Furthermore, myricitrin ameliorates cisplatin induced HK-2 cells damage and restores proper mitochondrial function by mitigating ferritinophagy mediated ferroptosis via NCOA4. Moreover, the pharmacological network analysis shows that myricitrin might possibly regulate the AKT and NF-κB pathways to provide anti-ferroptosis effects. Hence, myricitrin could be considered as a viable therapeutic intervention for treatment on cisplatin induced AKI.

## Data Availability

The original contributions presented in the study are included in the article/Supplementary material, further inquiries can be directed to the corresponding authors.
